# Prospective Visual Inspection of the Ventrum of Tongue (VIVOT) Vasculature Predicts the Presence of Esophageal Varices

**DOI:** 10.3390/gidisord6010017

**Published:** 2024-02-26

**Authors:** Martin Tobi, Monina Pascua, Rebecca Rodriguez, Yu-Xiao Yang, John Lieb, Douglas Weinstein, David E. Kaplan

**Affiliations:** 1 Department of Research and Development, Detroit John D. Dingle VAMC, Detroit, MI 48201, USA; 2 Gastroenterology Section, Corporal Michael J. Crescenz VA Medical Center, Philadelphia, PA 19104, USA; 3 The Oregon Clinic-Gastroenterology South, Oregon City, OR 97045, USA; 4 Division of Gastroenterology, Hepatology and Nutrition, Gainesville VAMC, University of Florida, Gainesville, FL 62308, USA; 5 Capital Health Gastroenterology Specialists, Pennington, NJ 08324, USA

**Keywords:** cirrhosis, esophageal varices, gastric varices, lingual, ventrum, tongue, endoscopy, fibrosis scans, vivot

## Abstract

Venous collateral shunting of blood from the splanchnic to systemic venous systems due to portal hypertension increases the pressure in the ventral lingual venous bed. We hypothesized that the appearance of sublingual varices evaluated by pre-endoscopy/bedside visual inspection of ventrum of tongue (VIVOT) might predict the presence of esophageal varices (EVs).

**Methods:**

To test this hypothesis, we prospectively enrolled patients with cirrhosis (CP) referred for EV screening for assessment of lingual vasculature after informed consent. Non-cirrhosis control patients were also enrolled.

**Methods:**

VIVOT was scored based on the presence of vessels > 2 mm and/or serpiginous veins. VIVOT scores were then correlated with endoscopic findings.

**Results:**

A total of 59 patients with cirrhosis (Group 1) were enrolled, as were 62 patients without cirrhosis (Group 2). Group 1 consisted of 100% male patients with mean age 59.5 ± 5.4 years; 39.0% were African American (AA). Group 2 consisted of 86% male patients, 59.0 ± 13 years and 53% AA. Among Group 1 patients, varices were present in 29% (16 esophageal and 3 gastric). There were no demographic differences among Group 1 patients with or without varices. Positive VIVOT scores were associated with EVs on endoscopy in 11 of 16 patients (sensitivity 68.75%). Positive VIVOT findings were present in 8 of 40 patients without EVs (specificity 80%). False-positive VIVOT scores were present in 6 of 62 non-cirrhotic controls. Overall, the positive predictive value among patients with cirrhosis was 59% with a negative predictive value of 84%.

**Conclusions:**

VIVOT has modest values in predicting EVs and should not be used alone to stratify patients for endoscopic evaluation when elastography and laboratory tests are available; however, its use in resource-limited settings to identify high-risk patients may be considered.

## 1. Introduction

Previous AASLD guidelines on portal hypertension recommended esophagogastroduodenoscopy (EGD) surveillance for large esophageal varices in patients not already on prophylaxis, with non-selective beta-blocker (NSBB) to identify varices needed to treat (VNT). However, large varices are identified in only ~15% of index EGDs and thus 85% of these procedures result in no intervention. Thus, there is interest in developing non-invasive approaches to identify patients at very low risk of VNT to exclude from endoscopic surveillance. The portal venous system does not have valves and therefore hypertension in the portal system readily transmits blood to the systemic venous system [1]. Non-invasive methods using elastography and laboratory testing exist for the prediction of EVs and have a PPV of 76–90% and NPV of 71–97% [2-8]. However, these technologies may be unavailable to many patients with less access to advanced care and acquiring such technology can be costly. We postulate that shunting of venous blood from the splanchnic to the systemic venous system through collateral venous connections increases pressure in the lingual venous bed of vessels, as previously suggested by others [1] and may co-occur with the appearance of EVs. Therefore, theoretically, a visual inspection of the ventrum of the tongue (VIVOT) could predict the presence of EV and gastric varices [9]. The aims and objectives of this study were to test the performance of VIVOT for predicting EVs via endoscopy.

## 2. Results

**Figure 1.**
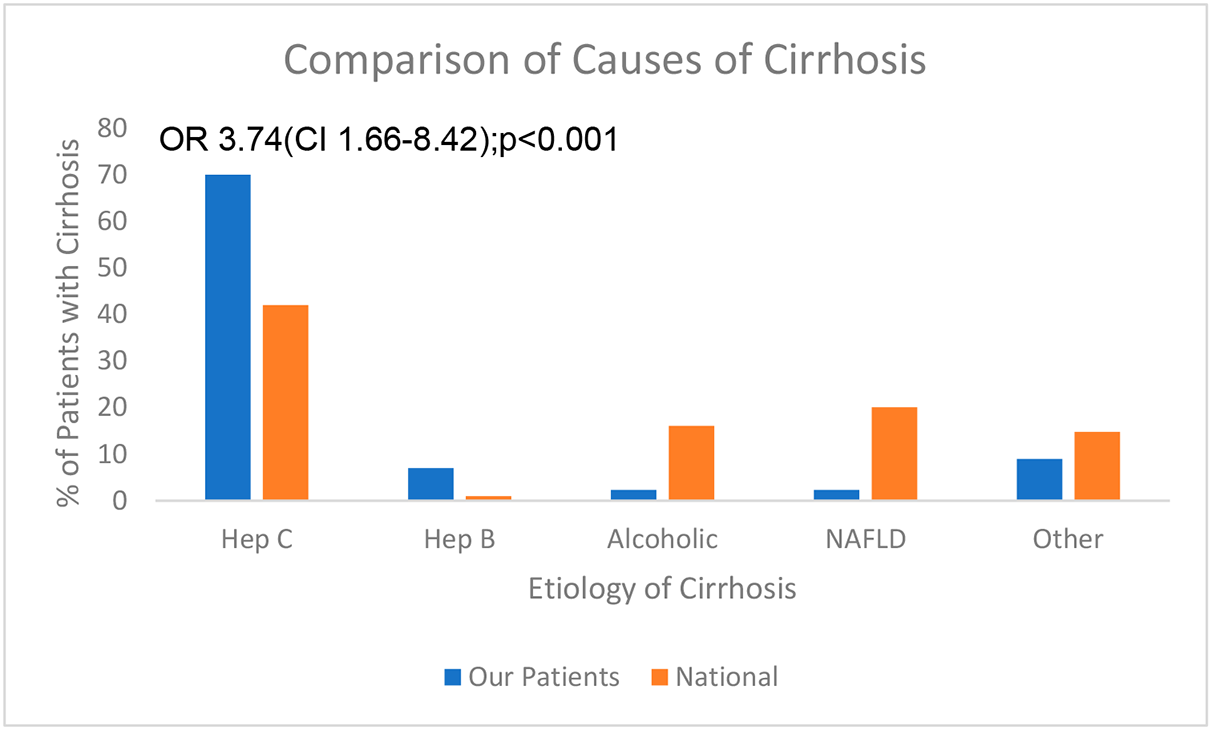
The approximate distribution of cirrhosis etiology.

We enrolled 131 patients but 10 were excluded (6 had no analyzable prediction data, 3 had incomplete consent forms, and 1 had a recent EGD). We were able to compare 59 CP (Group 1) with 62 controls (Group 2). Group 1 consisted of 100% male patients with a mean age of 59.5 years (sd ± 5.4) and 39.0% were of African American (AA) descent. The origin of cirrhosis was similar as in all veteran populations [10], namely 42% due to chronic hepatitis C, 16.1% due to alcoholic liver disease, 1% chronic hepatitis B, 20.1% NAFLD, and 14.8% other. Median Child–Turcotte–Pugh score was <6.5. Figure 1 illustrates the putative distribution of cirrhosis causation for the study patients versus published statistics in the Veterans Administration system.

The only significant difference in cirrhosis causation was in the chronic hepatitis C category, and we compared a large nationwide survey [10] with our much smaller study population. However, the linear regression analysis between the percentages of the two groups does not reach significance (r = 0.84; *p* = 0.07). The prevalence of varices in Group 1 was 29% (16 esophageal and 3 gastric). Group 2 comprised 86% male patients, 59.0 years (sd ± 13.0) and 53% AA, respectively. There were no significant age differences in age or race with respect to prevalence of varices within Group 1 (Table 1).

**Figure 2.**
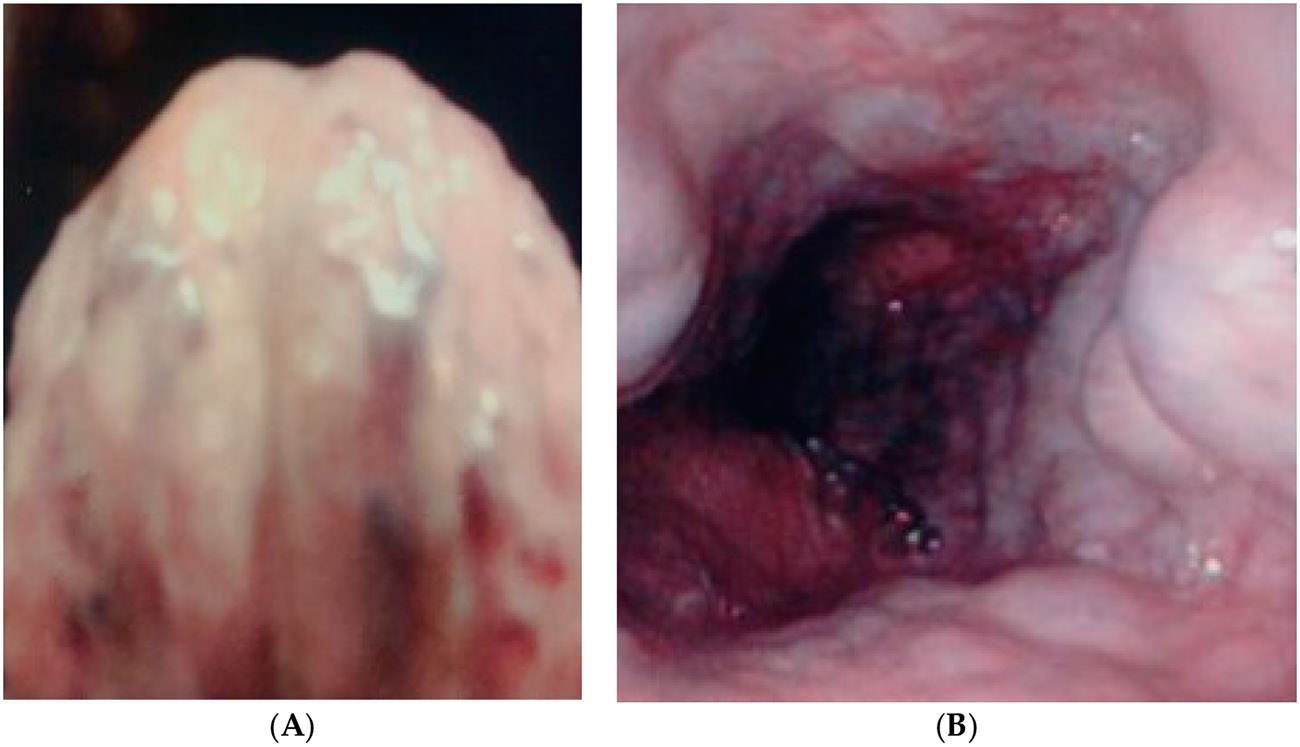
(**A**) Correct prediction in a 56-year-old African American male patient with known cirrhosis but not known to have had documented AVM. (**B**) shows large esophageal varices at endoscopy.

**Figure 3.**
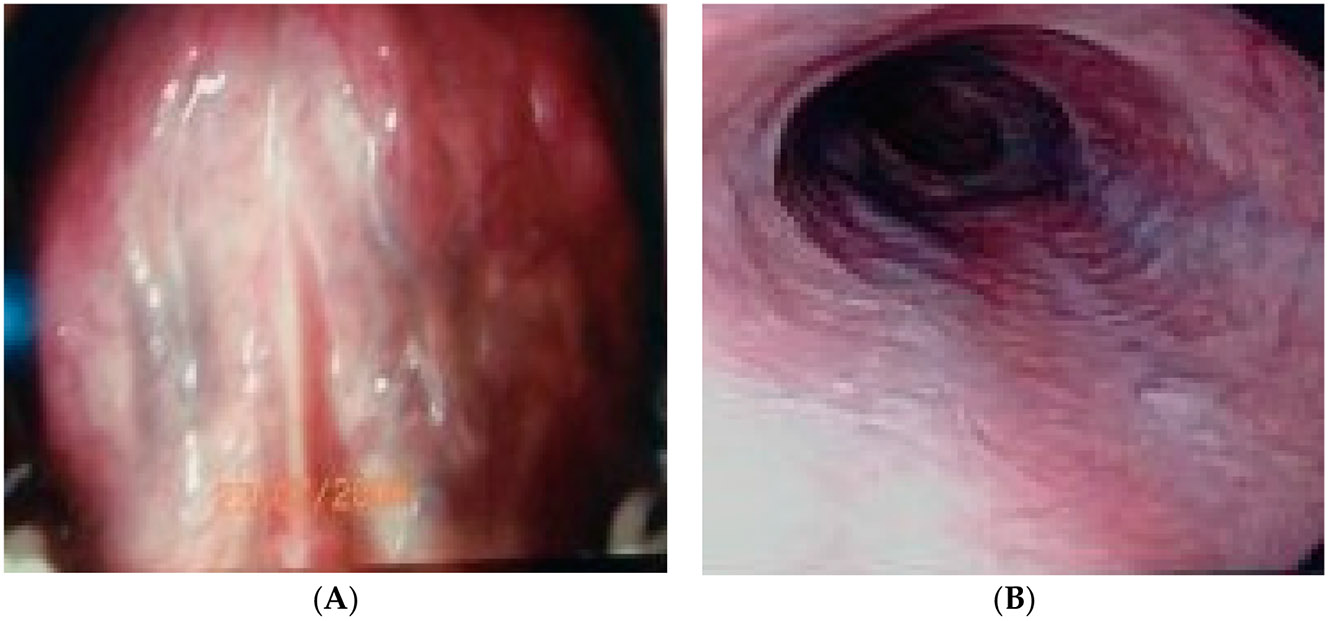
(**A,B**) Correct prediction in a 59-year-old African American male patient with known cirrhosis but who underwent variceal banding three years prior.

As in most studies in the Veterans Administration Healthcare System, over 95% tend to be male. Bedside/pre-endoscopy VIVOT was associated with the presence of esophageal varices on endoscopy (Figures 2 and 3) in 11 of 16 with and in 8 of 40 without esophageal varices (OR 6.3 Cl 1.8–22; *p* = 0.004); Odds ratio 15.6 [Cl 4.2–58]; *p* < 0.00005).

The VIVOT-predicted EVs on the basis of >2 mm veins, which extended almost to the tip of the tongue, was correctly predicted. See serpiginous, dilated veins towards tongue apex–ventral surface (Figure 2A). EVs (Figure 2B) on the right show more than three varices columns that were successfully banded at EGD.

Serpiginous veins >2 mm in diameter are seen on Figure 3A on the left. Two columns of grades 1–2 varices are seen on Figure 3B on the right. Although the banding was clearly significant at reducing the grade of the EV, they are still clearly visible.

**Figure 4.**
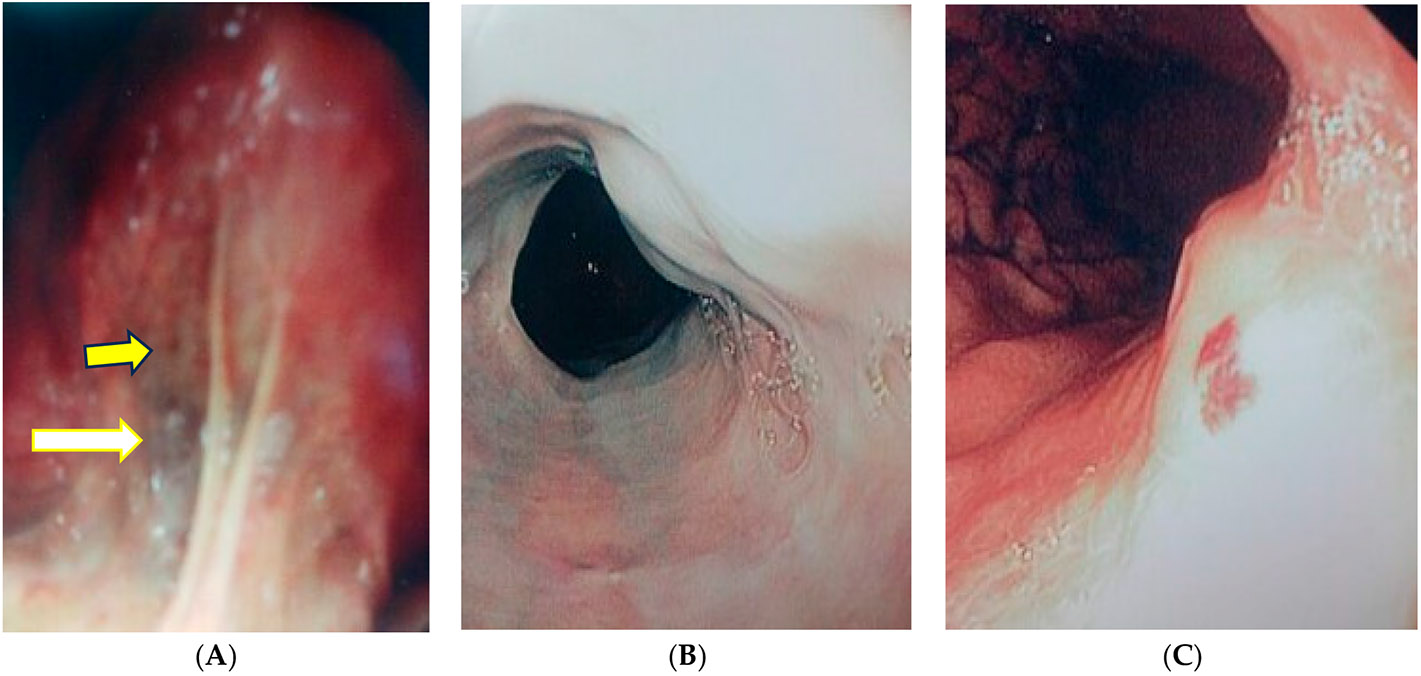
(**A–C**) Correct prediction in a 58-year-old Hispanic male patient with known cirrhosis.

**Figure 5.**
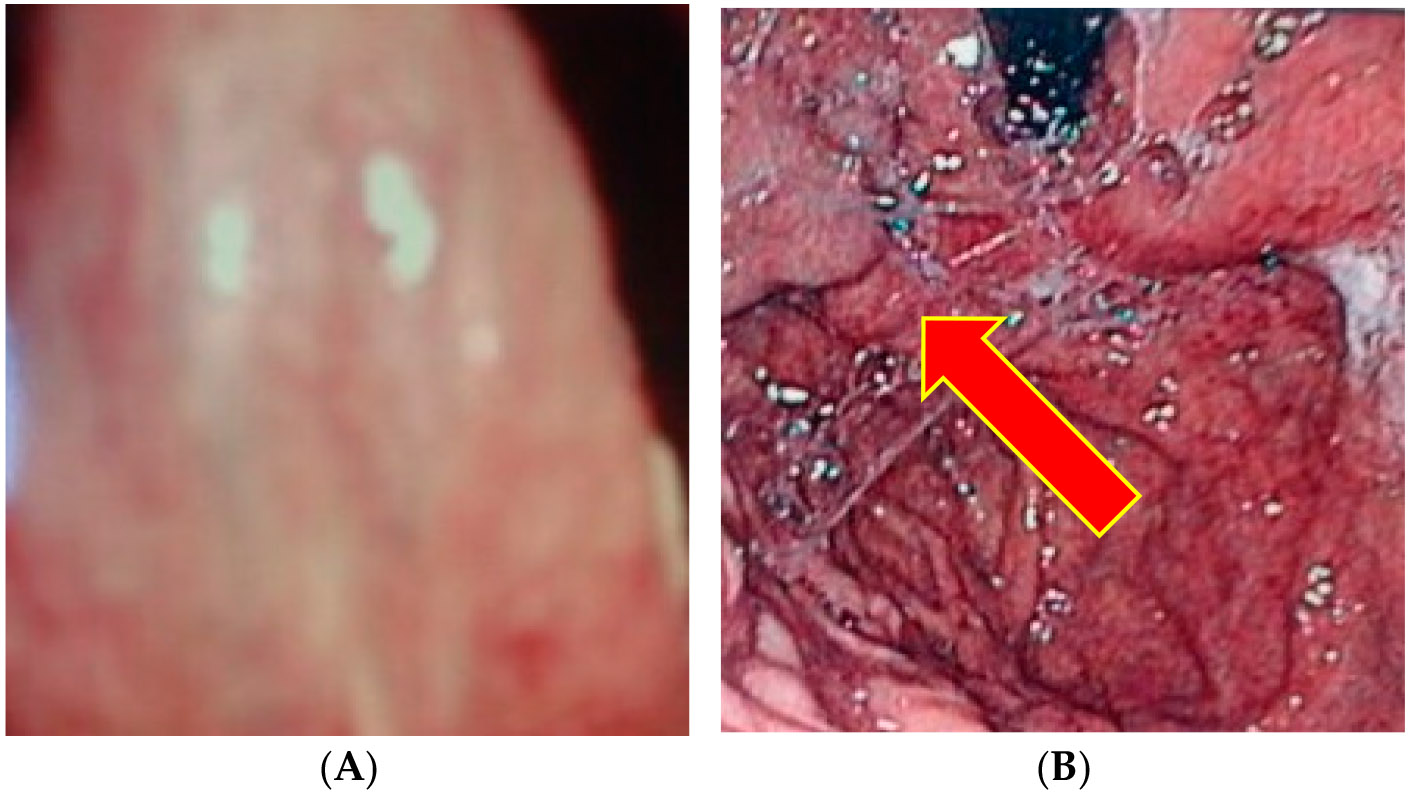
Correct prediction in a 58-year-old Caucasian male patient with known cirrhosis found to have gastric varices.

The VIVOT view shows dilated veins at the tongue base (open arrow) and a small arteriovenous malformation just above (solid arrow) in Figure 4A. A single column of grade 2 varices is seen in the middle panel (Figure 4B) and two contiguous, small non-bleeding, esophageal arteriovenous malformations are seen on the leftmost panel (Figure 4C). A depiction of gastric varices and VIVOT is seen in Figure 5

On the left panel is a depiction of an enlarged vein > 2 mm in diameter but linear in aspect as can be seen by the light reflex (Figure 5A). The right panel (Figure 5B) shows cardial varices near the retroflexed endoscope (arrow).

Among patients with cirrhosis, sensitivity of VIVOT was 69%, specificity 80%, PPV 59%, and NPV 84%. For the Group 2 VIVOT in whom tire prevalence of EVs was 0%, six patients had false-positive VIVOT scores, yielding a specificity of 90.1%. Thus, overall, sensitivity for EVs was 69%, specificity was 86.3%, PPV was 42%, and NPV was 95% (Table 2).

**Figure 6.**
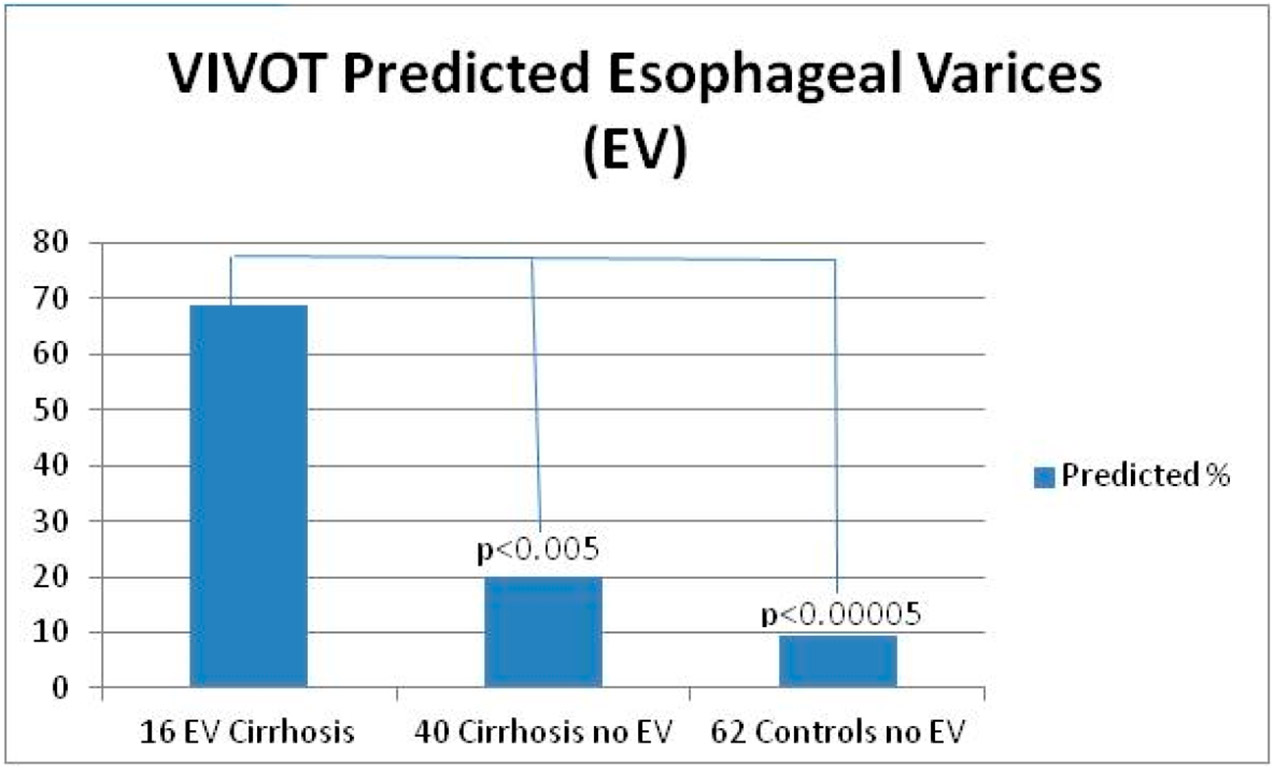
Percent of proportions and statistical outcomes in all groups.

VIVOT showed >2 mm veins, which extended almost to the tip of the tongue, as well as serpiginous, dilated veins towards tongue apex–ventral surface. Endoscopy confirmed EVs with more than three variceal columns that were successfully banded at EGD. In the example in Figure 5B, serpiginous veins > 2 mm in diameter were seen that correlated with grade 1–2 varices. In the example shown in Figure 4C, VIVOT showed dilated veins at the tongue base and a small arteriovenous malformation. A single column of grade 2 varices was observed as well as two contiguous, small non-bleeding, esophageal arteriovenous malformations. While AVMs found on VIVOT was not a major focus, they were of interest, and sought as per our protocol, and reflected a 50% sensitivity where EGD and colonoscopy were performed and might have been higher if endoscopy of the small bowel had also been performed. In the example shown in Figure 4B, an enlarged vein > 2 mm in diameter was observed along with gastrofundal varices near the retroflexed endoscope. Overall sensitivity of VIVOT for predicting EVs/GVs was close to 70% (Figure 6).

Figure 5 is a bar diagram showing a sensitivity of nearly 70% in the VIVOT prediction of EVs as opposed to patients with cirrhosis and controls without evidence of EVs at endoscopy.

## 3. Methods

### 3.1. Patient Recruitment

The protocol was approved by the Institutional Review Board at the Corporal Michael J. Crescenz VA Medical Center. Two cohorts of patients were enrolled: (1) patients with suspected clinically significant portal hypertension referred for EGD for variceal screening, and (2) patients without portal hypertension with various common indications. Inclusion and exclusion criteria are presented Table 3. Notable exclusion criteria included EGD in the prior 2 years or conditions/treatment liable to alter tongue appearance. Primary outcomes were the successful prediction of the presence of esophageal varices after VIVOT and secondary outcomes were the prediction of arteriovenous malformations after finding these on VIVOT.

### 3.2. Grading

**Figure 7.**
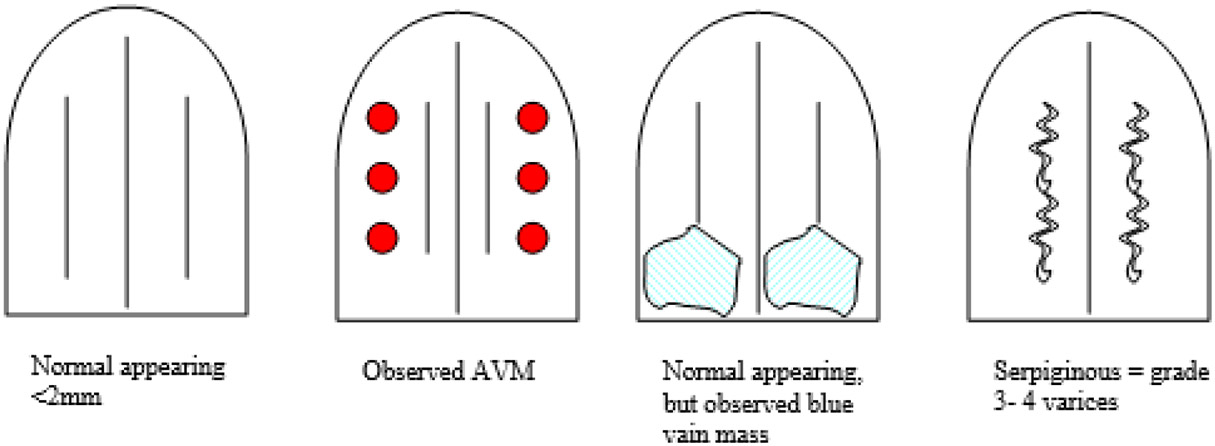
Guide to VIVOT topography.

After informed consent was obtained and prior to endoscopy, the endoscopist quantified features of the vascular patterns of the ventrum of the tongue, using the following criteria: vessel diameter > 2 mm; presence of serpiginous veins as demonstrated in Figure 7.

The ventrum of the tongue was photographed using a Sony DSC-W550 Super Steady Shot digital camera (Doral, FL, USA) by the research coordinator (RMR). Arteriovenous malformations (AVMs) were not a primary focus of this study but if coincidentally seen, these were noted (graded 1 to 4). The classification of VIVOT observations is shown in Table 4 as were the endoscopic findings. Gastric varices were characterized per the method of Sarin et al. [1]. Olympus GIF-XQ200 and GIF160 gastroscopes (Olympus America 2400 Ringwood Ave, San Jose, CA 95131, USA) were used; however, in cases of heavy hemorrhage, we had access to a double-channel endoscope (GIF-2TH-180) where the 2 channels could be used simultaneously (https://medical.olympusamerica.com/products/gastroscope/evisexera-ii-gif-2th180, accessed on 7 November 2023).

Website for Sarin classification: grades of gastric varices-Search (bing.com accessed on 7 November 2023). We used a simplification of this method originally for ease of operation, but it has since been superseded by newer classifications [9].

## 4. Statistical Analysis

Contingency tables measuring 2 × 2 were analyzed by chi-squared and/or Fisher’s exact test. Numeric variables were analyzed by linear correlation using the least squares method. Sensitivity, specificity, and positive predictive value were determined for various aspects of tongue morphology using standard formulae. We regarded *p* values of <0.05 as significant. Statistics were calculated online at VassarStats (URL, http://vassarstats.net/, accessed on 7 November 2023). The sample size calculation was based on a putative difference of at least 30% difference prediction between the comparison groups, where the estimated value of the smaller proportion in a group was estimated at 10%. Thus, with a β = 0.05 power and a 2-sided α = 0.02, we needed 69 in each group, for a total of 138, which proved to be optimal as can be seen in Section 2. The major statistical calculations are binary and not ordinal, based on proportional differences, and thus relies on non-parametric analysis, namely Fisher’s exact test. For determination of normality for ordinal data, we used the online Kolmogorov–Smirnov calculator (https://www.socscistatistics.com/tests/kolmogorov/, accessed on 5 February 2024).

## 5. Discussion

Bedside VIVOT predicted the presence of EVs in CP undergoing EGD. The sensitivity and specificity in this population compare modestly with more sophisticated and costly technologies whose sensitivities range from 74 to 90% and specificities from 75 to 86%. VIVOT may therefore be a low-cost method to prioritize CP with high risk for EVs for EGD and reduce unnecessary procedures [11]. Bedside VIVOT is extremely low-cost as it is performed as part of the physical exam. Also, VIVOT is easy to learn and could easily be implemented on a large scale.

There has been a paradigm shift in the detection treatment of esophageal and gastric varices (EVs and GVs). In the last decade, technology such as the FibroScan^™^ (Echosens, Atlanta, GA, USA) has revolutionized care for patients with cirrhosis [3,4] by streamlining the identification of clinically significant portal hypertension and reducing the need (Table 5) for esophagogastroduodenoscopy (EGD) as the primary means of detection [12]. However, the cost of this technology restricts access to developed countries.

Cirrhosis and resultant portal hypertension and other resulting disorders have now made liver disease the 11th leading cause of death [13] in the US (up from the 12th most common cause of death until recently), and most sufferers are unaware that they have the malady [7].

Portal hypertension is a final common pathway of many forms of liver disease and has consequences within the systemic circulation that allows for early identification and treatment of EVs by simple physical diagnostic means with a rapid and cost-free evaluation of blood vessel pattern on visual inspection of the of the ventrum of the tongue (VIVOT). VIVOT therefore may be an accessible, low-cost alternative for stratification of patients needing EGD to detect VNT.

Out-of-pocket expenses for an EGD exceeds USD 1000 (https://www.newchoicehealth.com/endoscopy/cost, accessed on 5 February 2024). The cost for VIVOT should not exceed the cost of a regular visit to a healthcare provider and should add nothing to the cost. However, we have not calculated the costs should VIVOT correctly identify the presence of varices and suggest that head-to-head studies [14] should be conducted with elastography and VIVOT.

Since the above guidelines were promulgated, new sets were advanced [12], including, by the AASLD [15]. In the former, endoscopy has 21 mentions and suggests that TE (transcutaneous elastography) should guide endoscopic interventions if the result is greater than 20 kPa or platelet count ≤ 150 × 10^9^/L in patients unable to take non-cardio-selective beta-blockers (NSBBs), graded C1 (Section 2.19). But for patients “avoiding” endoscopy, the approach is the same, of an unchanged D1 grade (Section 2.2 which is unchanged) and provides an “out” for those patients in the same category where the TE is ≤40 kPa (Section 2.2 C2 grading). In patients with compensated advanced chronic liver disease (cACLD) induced by hepatitis C, endoscopy would not be indicated when TE is ≤14 kPa and platelet count ≤ 150 × 10^9^/L, as mentioned above (Section 3.7 grade B1 new). In cACLD patients taking NSBBs where the primary cause of the cACLD has been successfully treated, endoscopy can be repeated at 1–2 years if TE is <25 kPa, and if no varices are obvious, NSBBs and can be discontinued. Follow-up times for repeat endoscopy after 2 years is not discussed in this section (grade C2 new, Section 3.9). Patients with compensated cirrhosis on NSBBs do not need endoscopy as this will not change management (new B2, Section 5.17) and neither will endoscopic grade D1, as new variceal ligation/compression/injection techniques which are changing therapy (grade D1 new, Section 5.18). In the interest of preventing decompensation in patients with ascites not taking NSBBs, endoscopy is indicated (grade B1 new, Section 7.3). The last recommendation relates to patients with documented portal vein thrombosis which have not recanalized within 6 months, who are encouraged to undergo endoscopy. Endoscopy should be repeated at 12 months and then at 2 years (grade B1, unchanged, Section 8.52). We did not consider endoscopy in acute hemorrhage. The 2019 AASLD guidelines [15] are similar but unique in describing gaps to be addressed.

Despite many and varying EV interventions, detection is an important first step and likely cost-effective with VIVOT. While it would be difficult to summarize the traditional Chinese literature regarding the role of tongue evaluation, there is a recent enlightening publication [16] where a nomogram of facial and sublingual features yields an indication of “blood stasis constitution”. Certainly, the detection of EVs by traditional Chinese medicine would be an exemplar of a static blood system with venous distention.

Finally, we also wish to clarify that we used the Fibroscan modality as a non-invasive reference, but in actuality, other non-invasive tests are available and fairly inexpensive—although less efficacious—whereby the best test was the platelet/spleen ratio test (it does require a splenic ultrasound, which costs USD 279 in Pennsylvania, where the study was conducted [https://cost.sidecarhealth.com/c/abdominal-ultrasound-cost], accessed on 5 February 2024), but has an AUC of 0.85 and was able to circumvent having to perform an EGD in 39% of patients with a 50% prevalence of EVs [17]. While the numbers of patients in our study conform to the population size of other modalities, there were technical difficulties regarding the quality of photographs of the tongue ventrum, which was quite challenging. The endoscope is adept at producing reasonable-quality reproductions but requesting a patient to elevate the tongue and hold it steady is daunting, particularly if the patient has hepatic encephalopathy, which most patients with the highest grade of EVs may have, especially with recent hemorrhage [14]. We do show an illustration (Figure 2) of arteriovenous malformations (AVMs) in this paper. We have also reported spider nevi in the stomach of CP [18] and that these might represent an under-identified cause of bleeding [19] and even mimic Dieulafoy-like bleeding [20], in that the pressure in these AVMs has been gauged at 90 mmHg, hinting at the potential for significant bleeding. Other authors have also reported tongue-related findings but only relative to the dorsum of the tongue [21-23]. Although we allowed for EVs of grades 1–4, we did not document grade 4 EVs, which have been replaced by the grade 3 or even the grade 2 system, despite most of the older classifications grading up to grade 4 EVs [24], published in 2016, years after the study concluded with four of six (one even with grade 5) including grade 4, with two having a grade 3 system. Furthermore, the study also began before the issuance of the 2007 AASLD guidelines on EVs grading using a three-tier system [25]. We collated the causes of cirrhosis in our patients and they showed only one significant difference from those reported in the Veterans Health Administration [26,27], and the management was similar [28]. Interestingly, the incidence of chronic hepatitis C in this study is estimated to be similar to that of the Detroit VAMC, NIPCON study [29] in patients with varices, at approximately 70 versus 64%, respectively. At this level, the linear regression calculation shows a similar distribution (r = 0.93; *p* < 0.021), suggesting that these two studies share a kinship in the prevalence of chronic hepatitis C. Generally speaking, the VHA has made significant inroads in the treatment of hepatitis C [30]. This will doubtlessly change the definition of the disease status of hepatitis C, with active versus eliminated categories [25].

Several limitations need to be acknowledged: First, the lack of blinding. The same endoscopists that rated the VIVOT also performed the endoscopy. Ideally, this should have been carried out by a non-rater, but we did not have the resources for extra endoscopists. However, the two endoscopists who performed most of the VIVOT predictions did not participate in our active hepatology division and therefore were independent raters, as the patients were unknown to them. Accordingly, endoscopy was their sole activity. Once the VIVOT score prediction was inscribed, it was given to the research coordinator who recorded in the database. Second, we did not compare the interobserver variability. Only after the photography from the endoscopy was available were the photographs catalogued and recorded in the database. Once made, predictions were final and no changes were allowed. Only one rater per patient performed the VIVOT evaluation. Third, VIVOT sensitivity and specificity, while not ideal and far lower from those of TE, are still interesting and merit further study.

## 6. Conclusions

In short, despite these important shortcomings, we believe VIVOT offers a low-cost method to screen for the presence of varices during physical examination. VIVOT could better direct the EV screening effort at a reduced cost, particularly relevant in emergent economies. We envisage that such a trial would be performed in countries where successful VIVOT predictions would represent an obvious medical advance.

## Figures and Tables

**Table 1.** Demographics and varices.

Parameter	Total	% Male	% Ethnicity AA	% Varices+	Mean Age * (yrs ± sd)
All Patients	131	93	59	56	59.5 ± 5.4
Cirrhosis Group 1	59	100	39	32 (16 EV:3 GV)	59.5 ± 5.4
Control Group 2	62	86	53	0	59.0 ± 13

yrs—years; sd—standard deviation; EVs—esophageal varices; GVs—gastric varices, noted but excluded from analysis limited to esophagus. * According to Kolmogorov–Smirnov ordinal data analysis, the age data conform to a normal distribution with identical values for both groups 1 and 2: *p* = 0.99; K-S D statistic = 0.069; skewness = −0.34; kurtosis = −0.5.

**Table 2.** VIVOT performance grading.

Parameter	% Sensitivity	% Specificity	% Positive Predictive Value	% Negative Predictive Value
Group 1	69	80	59	84
All Groups	69	86.3	42	95

**Table 3.** Inclusion and exclusion criteria.

Inclusion Criteria	Exclusion Criteria
All patients undergoing any of the following: Colonoscopy (for AVMs); Upper endoscopy; Enteroscopy and/or; Video capsule endoscopy; Liver cirrhosis diagnosed at the Corporal Michael J. Crescenz Veterans Affairs Medical Center.	Patients <18 years of age Patients unable to assent and/or consent for the procedure Patients with history of any of the following: Oral reconstructive surgery; Trauma to the oral cavity; Radiation to the oral cavity. EGD in the 2 years prior: Past gastrointestinal surgery (variceal-related); Except for any of the following: Cholecystectomy; Gastrectomy; Appendectomy; Colectomy. Patients with active and acute bleeding or debris obscuring mucosal detail.

**Table 4.** Data collection of observational study and expectations.

Grading (Photograph by Ruler)	Varices (Continuous)
*Sublingual varices* (also see figures)	0—normal-appearing veins < 2 mm 1—normal but serpiginous 2—veins > 2 mm 3—veins > 2 mm and serpiginous 4—dilated, serpiginous veins extending beyond borders of the frenulum continuous with varices
*Esophageal varices* (esophagus, stomach, duodenum, jejunum, and ileum)	0—normal-appearing veins 1—normal ≥1 mm but <1 cm 2—thin-walled, distended veins 3—varicosities, distended >1 cm 4—distended approximately 2 cm
*Gastric varices GOV1 and GOV2, IGV1 and IGV2 by Sarin’s Method* (by location) *Duodenum*	1—esophageal and gastric varices that extend to the lesser curvature 2—esophageal and gastric varices that extend to the greater curvature 1—isolated gastric (IGV1) alone, confined to the fundus 2—isolated gastric or duodenal varices (IGV2) 0—none 1—present regardless of size

**Table 5.** Instances where VIVOT may replace endoscopy according to the Baveno VII Criteria (12).

Patients Use of NSBB	Elastography Criteria/Ascites	Platelet Criteria	Endoscopy (E) Indicated
Unable	Greater than 20 kPa/None	≤150 × 10^9^/L	Yes (C1, Section 2.19)
Unable but avoiding	Greater than 20 kPa/None	≤150 × 10^9^/L	Unchanged (D1, Section E 2.2)
Unable but avoiding	Less than 40 kPa/None	≤150 × 10^9^/L	No E (C2, Section E 2.2)
Hepatitis comp	Less than 14 kPa	≤150 × 10^9^/L	No E (B1, Section 3.7)
Able, treated	Less than 25 kPa (E 1–2 years)	N/A	E-cACLD E-ve; dcNSBB (C2, Section 3.9)
Able, comp	N/A	N/A	No E (B2, Section 5.7)
No NSBB/ascites	N/A	N/A	E-yes (B2, Section 7.3)

Abbreviations: NSBB—non-cardio-selective beta-blocker; cACLD—compensated (comp) advanced chronic liver disease (cACLD); E-ve—endoscopy negative; dc—discontinue. Issue of documented portal vein thrombosis with no recanalization at 6 months’ endoscopy surveillance is suggested (B1, Section 8.52). N/A = not applicable.

## Data Availability

This will be dictated by US Government Policy.
